# The Reconstruction of Condition-Specific Transcriptional Modules Provides New Insights in the Evolution of Yeast AP-1 Proteins

**DOI:** 10.1371/journal.pone.0020924

**Published:** 2011-06-09

**Authors:** Christel Goudot, Catherine Etchebest, Frédéric Devaux, Gaëlle Lelandais

**Affiliations:** 1 Dynamique des Structures et Interactions des Macromolécules Biologiques (DSIMB), INSERM, U665, Paris, France; 2 Université Paris Diderot, Sorbonne Paris Cité, UMR-S665, Paris, France; 3 INTS, Paris, France; 4 Laboratoire de Génomique des Microorganismes, UMR7238 CNRS, Université Pierre et Marie Curie, Paris, France; Institut de Genetique et Microbiologie, France

## Abstract

AP-1 proteins are transcription factors (TFs) that belong to the basic leucine zipper family, one of the largest families of TFs in eukaryotic cells. Despite high homology between their DNA binding domains, these proteins are able to recognize diverse DNA motifs. In yeasts, these motifs are referred as YRE (Yap Response Element) and are either seven (YRE-Overlap) or eight (YRE-Adjacent) base pair long. It has been proposed that the AP-1 DNA binding motif preference relies on a single change in the amino acid sequence of the yeast AP-1 TFs (an arginine in the YRE-O binding factors being replaced by a lysine in the YRE-A binding Yaps). We developed a computational approach to infer condition-specific transcriptional modules associated to the orthologous AP-1 protein Yap1p, Cgap1p and Cap1p, in three yeast species: the model yeast *Saccharomyces cerevisiae* and two pathogenic species *Candida glabrata* and *Candida albicans*. Exploitation of these modules in terms of predictions of the protein/DNA regulatory interactions changed our vision of AP-1 protein evolution. *Cis*-regulatory motif analyses revealed the presence of a conserved adenine in 5′ position of the canonical YRE sites. While Yap1p, Cgap1p and Cap1p shared a remarkably low number of target genes, an impressive conservation was observed in the YRE sequences identified by Yap1p and Cap1p. In *Candida glabrata*, we found that Cgap1p, unlike Yap1p and Cap1p, recognizes YRE-O and YRE-A motifs. These findings were supported by structural data available for the transcription factor Pap1p (*Schizosaccharomyces pombe*). Thus, whereas arginine and lysine substitutions in Cgap1p and Yap1p proteins were reported as responsible for a specific YRE-O or YRE-A preference, our analyses rather suggest that the ancestral yeast AP-1 protein could recognize both YRE-O and YRE-A motifs and that the arginine/lysine exchange is not the only determinant of the specialization of modern Yaps for one motif or another.

## Introduction

Studies of the evolution of transcriptional regulatory networks, which control all phenotypic features, critically depend on the ability to accurately characterize and compare transcriptional modules (TMs) in several different related species. A TM can be defined as the set of genes whose transcription is modulated by a common transcription factor (TF). The characterization of TMs raises challenging questions regarding both the choice of the experimental datasets and the bioinformatics methodologies to examine these data. For instance, expression patterns of genes measured with genome-wide technologies are often analyzed applying clustering approaches that identify groups of co-expressed genes [1], [2]. Clustering on the basis of expression data alone is highly efficient to identify functionally related groups of genes [3], [4], [5], but it only gives an indirect access to the TFs that underlie gene co-expression. To enhance the reconstruction of TMs other data types have to be used. Transcriptome analyses of mutants, in which the gene coding for a particular TF has been deleted, gives valuable information concerning the genes for which transcription depends, directly or indirectly, on the presence of this TF [6], [7]. Additionally, protein/DNA interaction data obtained using ChIP-chip or ChIP-seq technologies allow the identification of the set of genes whose promoter sequences directly bind a particular TF *in vivo* [8]. In this context, an optimal approach is to combine several types of experimental data for the same TF in different species. One clear challenge therefore concerns the development of methodologies for module discovery based on heterogeneous information [9], [10], [11], [12]. In this study, we aimed at optimizing simultaneously *(i)* the discovery and *(ii)* the cross-species comparisons of TMs. For that, we developed an original approach that relied on two main points. First, multiple biological data sources and bioinformatics methodologies were combined using an integrative procedure whose objective was to minimize the risk to select false positive genes in the final TMs. Second, as one TF could control different sets of genes depending on the cell state or the environmental conditions, we used only data obtained in a specific experimental condition, identical in all the species examined. We applied this rationale to the analysis of AP-1 proteins in three different yeasts: the model yeast *Saccharomyces cerevisiae* (*S. cerevisiae*) and two pathogenic species *Candida glabrata* (*C. glabrata*) and *Candida albicans* (*C. albicans*).

AP-1 proteins belong to the basic leucine zipper (bZIP) family that represents one of the largest families of TFs in eukaryotic cells. They have the particularity to bind DNA as dimers (homo- or hetero-dimers), which interact through repeats of leucine residues every seven amino acids to form a coiled coil region [13]. Two flanking α-helices constitute the basic region, which contacts DNA [13]. In this study, we focused on the AP-1 proteins Yap1p (in *S. cerevisiae*), Cgap1p (in *C. glabrata*) and Cap1p (in *C. albicans*). These three proteins are functional homologous TFs [14], [15], [16], and are the central regulators of the response to oxidative stress in their respective species [6], [16], [17]. They also play a significant role in multidrug resistance [14], [16], [18]. They control the expression of many enzymes involved in redox homeostasis, but also genes encoding multidrug transporters. In the model yeast *S. cerevisiae*, the DNA binding motifs recognized by Yap1p have been extensively studied. Six motifs have been experimentally characterized: TTACTAA [19], TTACTCA [20], TTAGTCA [19], TTACAAA [20], [21], TGACAAA [20] and TGACTCA [22]. They are referred to as Yap Response Element (YRE) and share common properties: *(i)* these motifs are seven or eight base pairs long, *(ii)* they are palindromic or pseudo-palindromic sequences starting with a TTA or a TGA triplet and *(iii)* they have a central (C/G) base pair. Kuo *et al.* [23] recently extended this definition of YREs by describing the canonical YRE motifs as two TTAC “half sites” positioned either in an adjacent (TTACGTAA referred as YRE-A) or in an overlapping fashion (TTA(C/G)TAA referred as YRE-O). As mentioned above, Yap1p recognizes motifs derived from the YRE-O subtype, with a clear preference for the perfect YRE-O consensus TTA(C/G)TAA [19]. In *C. albicans*, the canonical YRE-O has also been proposed as the Cap1p preferred DNA binding motif [16], [24], [25], [26]. Intriguingly in *C. glabrata*, Cgap1p DNA binding properties appears to have changed. Using transcriptome data and directed mutagenesis, we demonstrated in a previous study [21] that TTACAAA, a YRE-O variant that is rarely found in Yap1p target genes, acts as a significant Cgap1p response element. Kuo *et al.* [23] proposed that, due to a single mutation in its DNA binding domain, Cgap1p binds exclusively YRE-A motifs.

Like many other TFs in yeasts (for instance Ste12p and Tec1p [27]), Yap1p, Cgap1p and Cap1p do not act in a stereotypical manner. Their activity can vary qualitatively and quantitatively, depending on the origin of the oxidative stress encountered by the cells [28]. In this study, we therefore focused our multispecies comparative analyses on the AP-1 TMs involved in the response of the cells to a particular environmental stimulation, *i.e.* the presence of the antifungal drug benomyl. Benomyl was chosen because it was the only AP-1-activating agent for which sufficient, comparable experimental information was available in the three yeast species. Using transcriptome analyses of the genomic response to benomyl induced-stress in both wild type and AP-1-deleted strains, together with Chromatin ImmunoPrecipitation on Chip (ChIP-chip) experiments, we defined the Yap1p, Cgap1p and Cap1p benomyl-specific TMs (bTMs). Cross-species comparisons of the AP-1 bTMs showed that bTM-genes shared a surprisingly few orthologous and homologous relationships. Subsequent analyses of the *cis*-regulatory motifs located in the promoters of genes in each bTM brought important new information regarding the DNA binding properties of the AP-1 TFs. First, our analyses suggested that, when they interact with DNA, the yeast AP-1 proteins cover a larger DNA fragment than strictly the TTA•TAA half sites, with a conserved adenine located in 5′ of the YREs. Second, YRE-O motifs were highly conserved between *S. cerevisiae* and *C. albicans* species, whereas significant divergences were observed in *C. glabrata*. In particular, our data strongly suggested that Cgap1p is able to recognize both YRE-O and YRE-A motifs. This hypothesis is supported by structural data available on the Pap1p TF, an AP-1 protein in the yeast *Schizosaccharomyces pombe* (*S. pombe*), which is also able to recognize YRE-O and YRE-A *cis*-regulatory motifs.

## Results

### Integration of multiple data sources for the reconstruction of condition-specific transcriptional modules

**Figure 1:**
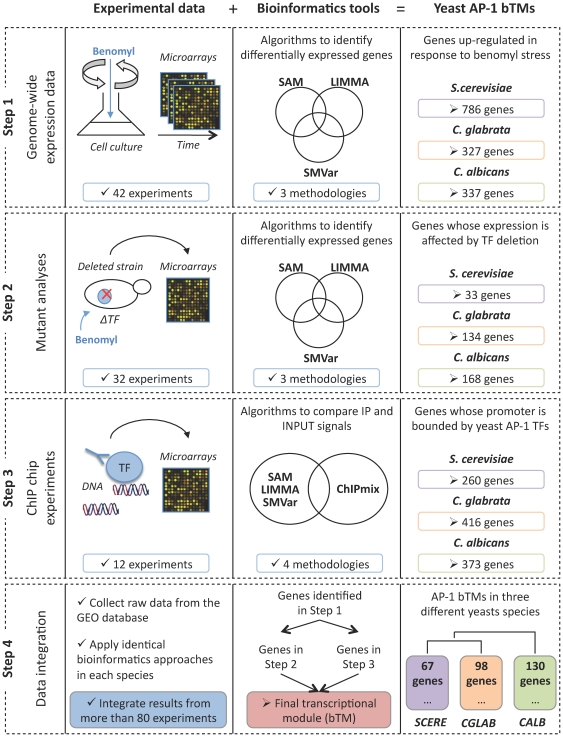
Reconstruction of the yeast AP-1 benomyl-specific transcriptional modules (bTMs) in species *S. cerevisiae*, *C. glabrata* and *C. albicans*. Three different sources of genome-wide experimental datasets (expression data, mutant analyses and ChIP-chip experiments) were collected from the literature and successively analyzed using several bioinformatics tools. In each yeast species (*S. cerevisiae*, *C. glabrata* and *C. albicans*) the same procedure, divided into four independent steps, was applied. Step 1 consisted in identifying genes whose expression was up regulated in response to benomyl induced-stress. Results arising from 42 microarray experiments were analyzed using a combination of 3 different algorithms SAM, LIMMA and SMVar (see Materials and Methods). 786, 327 and 337 genes were respectively selected in *S. cerevisiae*, *C. glabrata* and *C. albicans*. Step 2 consisted in identifying genes whose expression in response to benomyl induced-stress was affected by the deletion of genes coding TFs Yap1p (in *S. cerevisiae*), Cgap1p (in *C. glabrata*) or Cap1p (in *C. albicans*). 32 microarray experiments were analyzed using the algorithm SAM, LIMMA and SMVar (see Material and Methods) and 33, 134 and 168 genes were identified in *S. cerevisiae*, *C. glabrata* and *C. albicans* genomes, respectively. Step 3 consisted in identifying genes whose promoter interacted *in vivo* with TFs Yap1p, Cgap1p or Cap1p. Data obtained with ChIP chip technologies (12 experiments) were analyzed combining SAM, LIMMA and SMVar algorithms together with ChIPmix program. 260, 416 and 373 genes were thus identified respectively in *S. cerevisiae*, *C. glabrata* and *C. albicans*. Finally, Step 4 consisted in data integration. For that results obtained in Step 1, 2, and 3 were combined using the following rule: to be conserved in the final AP-1 bTM a gene had to be selected in “Step 1 and Step 2” or in “Step 1 and Step 3”. In *S. cerevisiae* (SCERE) the Yap1 bTM therefore comprised 67 genes, in *C. glabrata* (CGLAB) the Cgap1p bTM comprised 98 genes, and finally in *C. albicans* (CALB) the Cap1p bTM comprised 130 genes. All together, we combined in this analysis experimental results arising from more than 80 individual microarray experiments applying different bioinformatics methodologies. The predictive strength of the strategy is based on the combined constraints that arise from the use of multiple biological and bioinformatics data sources.

We designed an integrative framework (Figure 1) to identify the sets of genes for which transcription was activated by Yap1p (in *S. cerevisiae*), Cgap1p (in *C. glabrata*) and Cap1p (in *C. albicans*) in response to a specific physiological stimulation, *i.e.* cell treatment by the antifungal drug benomyl. This framework combined three different sources of genome-wide experimental data, together with several effective bioinformatics approaches to analyze them. In a first step, our aim was to characterize sets of benomyl responsive genes in all three species. For that, we used published microarray datasets quantifying the transcriptome responses of the yeasts *S. cerevisiae*, *C. glabrata* and *C. albicans* to similar doses of benomyl for similar time periods [16], [21] (see Materials and Methods). As each dataset came from different laboratories using different methodologies, we started our analysis from the initial raw data and applied in each species the same procedure for identifying genes whose transcription was significantly modified after benomyl addition (see Text S1 for a comparison of the list of genes defined in this study with these originally published). We used a combination of three different algorithms: SAM [29], LIMMA [30] and SMVar [31] (see Materials and Methods). As a result, 786 genes were identified as being significantly up regulated in *S. cerevisiae*, 327 genes in *C. glabrata* and 337 genes in *C. albicans* (Figure 1, Step 1). In a second step, we specifically highlighted the genes whose benomyl induction was dependent on Yap1p, Cgap1p or Cap1p. We analyzed transcriptome data comparing the benomyl response of *ΔYAP1*, *ΔCgAP1* and *ΔCAP1* strains with the response of the corresponding wild type strains (see Materials and Methods). The combination of the algorithms SAM, LIMMA and SMVar mentioned above allowed us to identify 33 genes as being Yap1p-dependent in *S. cerevisiae*, 134 genes as being Cgap1p-dependent in *C. glabrata* and 168 genes as being Cap1p-dependent in *C. albicans* (Figure 1, Step 2). In a third step, we analyzed ChIP-chip experiments performed for TFs Yap1p, Cgap1p and Cap1p to identify the genes that were directly bound by these proteins (see Materials and Methods). As for transcriptome data, we re-analyzed the raw ChIP-chip data by combining SAM, LIMMA and SMVar algorithms with the ChIPmix algorithm [32] (see Materials and Methods). We found 260 genes whose promoters were associated to Yap1p in *S. cerevisiae*, 416 genes whose promoters were associated to Cgap1p in *C. glabrata*, and 373 genes whose promoters were associated with Cap1p in *C. albicans* (Figure 1, Step 3). The results obtained in Step 1, 2 and 3 were finally integrated (Figure 1, Step 4). We defined as members of the final AP-1 bTMs (for benomyl-specific Transcriptional Modules), genes that were *(i)* up regulated by benomyl (Step1) and (*ii*) sensitive to the deletion of the corresponding AP-1 TF (Step 2) or directly bound in promoter by this TF (Step 3). Using these criteria, the Yap1p bTM comprised 67 genes in *S. cerevisiae*, the Cgap1p bTM comprised 98 genes in *C. glabrata*, and the Cap1p bTM comprised 130 genes in *C. albicans*. Complete list of genes in each bTM together with their corresponding functional description can be found in Dataset S1. Therefore the bTMs described in this study had the particularity *(i)* to be focused on the AP-1 responsive genes in benomyl stress-induced conditions (genes regulated by AP-1 TFs in other conditions were not considered), and *(ii)* to include only genes for which different types of experimental evidences were available for interactions with Yap1p, Cgap1p or Cap1p. This last criterion allowed us to minimize the false positive error rate, *i.e.* genes that could be identified as AP-1 TF target genes only due to the background inherent to one particular technique (see also Text S2 for a detailed justification of these selection procedure).

### Sequence orthology between genes only slightly reflect functional similarities between AP-1 benomyl-specific transcriptional modules

**Figure 2:**
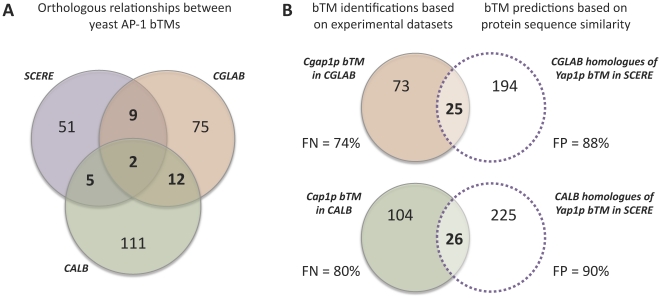
Cross-species comparison of the *S. cerevisiae*, *C. glabrata* and *C. albicans* bTMs based on sequence orthology and homology. (A) Yeast AP-1 bTMs were defined using the general protocol presented in Figure 1. They are represented here using a Venn diagram with the following color code: purple circle for Yap1p bTM (SCERE), orange circle for Cgap1p bTM (CGLAB) and green circle for Cap1p bTM (CALB). Overlaps between bTMs represent the number of orthologous relationships (inferred with the INPARANOID algorithm, see Materials and Methods) between them. Only 11 orthologous genes were thus identified between the SCERE and CGLAB AP-1 bTMs (16%), 7 between the SCERE and CALB AP-1 bTMs (10%) and 14 between the CGLAB and the CALB AP-1 bTMs (14%). Considering the global amount of orthologous genes between the three species (more than 60%), these values were surprisingly low and suggested that in yeasts, there exist functional similarities between proteins that are not reflected in sequence orthology. (B) Comparison between the Cgap1p (in *C. glabrata*) and the Cap1p (in *C. albicans*) bTMs identified based on experimental datasets, and the bTMs predicted based on protein sequence similarity with the Yap1p (in *S. cerevisiae*) bTM, *i.e.* functional annotation transfer from the model yeast *S. cerevisiae* to the *Candida* species. The original Cgap1p and Cap1p bTMs are represented using respectively orange and green circles, whereas the predicted bTMs are shown with circles surrounding by purple dashed lines. The predicted bTMs were obtained searching in *Candida* genomes for homologous proteins with the Yap1p bTM using the BLAST algorithm (see Materials and Methods). Overlaps between original and predicted bTMs represent the number of genes in common. Considering the *Candida* bTMs identified using experimental datasets as a reference, false positive (FP) and false negative (FN) rates associated to the bTMs predictions were calculated and are shown here. In each species, FN and FP represent important error rates (more than 70%), if one tries to defined AP-1 bTMs in *Candida* species directly transferring information from the well-studied *S. cerevisiae* species.

Orthology defines the relationship between genes in different species that originate from a single gene in the last ancestor of these species [33], [34], [35]. Orthologous genes are therefore most likely to have similar functions and may exhibit conserved regulatory controls. Considering that the TFs Yap1p, Cgap1p and Cap1p are functional homologues [14], [15], [16], which play similar physiological roles in the cell [6], [16], [17], one could expect that the AP-1 bTMs defined above would include mainly orthologous genes. To test this hypothesis, we performed a cross-species comparison of the bTMs using orthology assignements. We applied the INPARANOID algorithm [36] comparing all the protein sequences of the three yeast species (see Materials and Methods). Orthologous links were inferred for 80% of the genes comparing the *S. cerevisiae* and *C. glabrata* genomes, 61% of the genes comparing the *S. cerevisiae* and *C. albicans* genomes, and 63% of the genes comparing the *C. glabrata* and *C. albicans* genomes. These results were coherent with the phylogeny of the yeast species analyzed here, *i.e. C. glabrata* being more closely related to *S. cerevisiae* than *C. albicans* is. Then, we determined whether orthologous genes were present in each of the three AP-1 bTMs. Strikingly we found only 11 orthologous links between the *S. cerevisiae* and *C. glabrata* AP-1 bTMs (16%), 7 between the *S. cerevisiae* and *C. albicans* AP-1 bTMs (10%) and 14 between the *C. glabrata* and the *C. albicans* AP-1 bTMs (14%) (Figure 2A). Assuming that the definition of orthology links obtained with INPARANOID may be too stringent, we next applied the BLAST program searching for “homologous proteins” between the three yeast genomes (see Material and Methods). For the 67 genes that belong to the *S. cerevisiae* Yap1p bTM, we therefore identified 219 and 251 homologous proteins in *C. glabrata* and *C. albicans* genomes, respectively. Complete list of genes can be found in Dataset S2. Again, from all these genes only a small subset were included in the *C. glabrata* and *C. albicans* AP-1 bTMs defined using experimental information (respectively 25 and 26, Figure 2B). This represented 37% and 39% of the 67 *S. cerevisiae* input genes. In agreement with previous observations [23], these overlaps were still statistically significant (p-values<10^−10^) compared to a random model in which the three bTMs would have been completely shuffled through evolution. But on the other hand, these data were also clearly different from a full conservation model. It indicated that the functioning of Yap1p, Cgap1p and Cap1p TFs during the transcriptional response to benomyl stress has been significantly rewired. Noteworthy, this also meant that the classical approach that consists in directly transferring functional annotations from well-studied organisms (like *S. cerevisiae*) to the newly sequence species (like *Candida* species) using only protein sequence homology would have led, in case of yeast AP-1 bTMs, to a high rate of false positives and false negatives genes (higher than 70%, Figure 2B).

### *De novo cis*-regulatory motif predictions refine the evolution of Yap Response Elements

**Figure 3:**
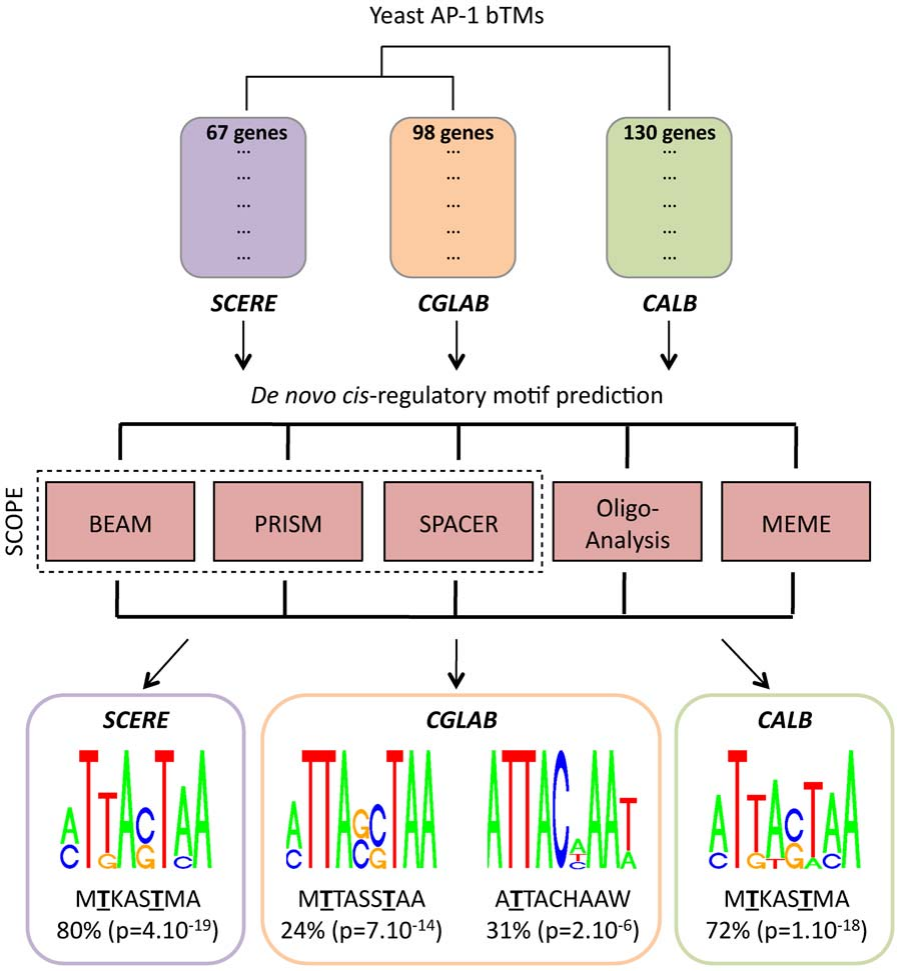
Identification of *cis*-regulatory motifs in promoter sequences of AP-1 bTM genes. Yeast AP-1 bTMs were characterized using the procedure presented in Figure 1. They are represented here using the following color code: Yap1p bTM in *S. cerevisiae* (SCERE) in purple, Cgap1p bTM in *C. glabrata* (CGLAB) in orange, Cap1p bTM in *C. albicans* in green. Promoter sequences of genes were analyzing using a combination of five different algorithms (BEAM, PRISM, SPACER, Oligo-Analysis and MEME) and applying a filter procedure to select the most significant motifs (see Material and Methods and Text S3). 12 motifs were identified in SCERE, 7 motifs in CGLAB and 8 motifs in CALB. They are presented in Text S4. In each species, these motifs were combined and consensus sequences are shown here (SeqLogo representations). A unique consensus MTKASTMA was observed in promoters of SCERE and CALB genes and two consensuses (MTTASSTAA, ATTACHAAW) were observed in promoters of CGLAB genes (where M designates A or C, K designates G or T, S designates C or G and W designates A or T). Percentages of genes in each AP-1 bTMs that exhibit those consensuses are indicated below the SeqLogo representations, with the associated enrichment p-value (see Materials and Methods). Highly conserved positions between the consensuses are underlined. They are predicted to strongly interact with the TF DNA binding domain, based on structural inspection of the Pap1p/DNA complex (see Figure 4).

Compared with previous works, the yeast AP-1 bTMs defined in this study had the originality to arise from the combination of condition-specific transcriptome experiments and ChIP-chip data. Assuming that this approach resulted in a physiologically more relevant and accurate view of the yeast AP-1 target genes, we next investigated the regulatory mechanisms that guide the functioning of the yeast AP-1 proteins, analyzing *cis*-regulatory motifs in the promoter sequences of bTM-genes. We used an original procedure that combined five different motif discovery algorithms: BEAM [37], PRISM [38] and SPACER [39] (combined in the SCOPE program [40]), Oligo-Analysis [41] and MEME [42]. These algorithms were chosen because they use different theoretical background and hence were each designed to identify a particular class of motifs (short non-degenerate motifs, short-degenerate motifs, long highly degenerate motifs, motifs with non-contiguous critical residues, etc.). Promoter sequences of genes in yeast AP-1 bTMs were analyzed searching for potential regulatory motifs (see Materials and Methods). To combine and filter the results obtained with each algorithm we applied the global procedure illustrated in Text S3. To summarize, the approach consisted in *(i)* collecting all the motifs proposed by each algorithm, *(ii)* removing irrelevant motifs that were too short for being specifically recognized by AP-1 proteins (<7 base pairs) and motifs with more than three uncharacterized positions, *(iii)* ordering the remaining motifs according to their enrichment p-values and conserving the most significant ones, *i.e.* with a p-value<10^−5^, and *(iv)* selecting the motifs that agreed steps *(i)* to *(iii)* and that were identified with at least two different algorithms. As a result, 12 motifs were identified in *S. cerevisiae*, 7 motifs in *C. glabrata* and 8 motifs in *C. albicans*. Detailed motif information can be found in Text S4 and the corresponding consensus sequences together with sequence logos are presented in Figure 3. Interestingly, a unique consensus sequence MTKASTMA was enriched in promoter sequences of genes in both the Yap1p and Cap1p bTMs. The corresponding p-values were highly significant, at 4.10^−19^ (Yap1p bTM in *S. cerevisiae*) and 1.10^−18^ (Cap1p bTM in *C. albicans*). Notably this sequence *(i)* was present in more than 70% of the promoters of Yap1p- and Cap1p-dependent genes, *(ii)* included YRE-O motifs with in particular, the palindrome sequence TTA(C/G)TAA characterized previously as being the main benomyl response element (BRE) in these two species [16], [19], and *(iii)* exhibited a supplementary adenine (or to a less extend a cytosine) in 5′ position. In *C. glabrata*, the identified motifs could be combined into two different consensuses MTTASSTAA (p-value = 7.10^−14^) and ATTACHAAW (p-value = 2.10^−6^). These consensuses were 9 base pair long with again, A or C in the 5′ position. The MTTASSTAA consensus could be related to the YRE-A motifs, which were recently proposed to be the main Cgap1p DNA binding sequences [23]. Strikingly enough, this motif was present in only 24% of the promoters of Cgap1p-dependent genes. The second consensus ATTACHAAW could be related to YRE-O motifs and included the TTACAAA sequence, which was previously demonstrated to act as a BRE in *C. glabrata* [21]. This consensus sequence was found in 31% of Cgap1p-dependant gene promoters. All together, the MTTASSTAA and ATTACHAAW motifs were present in half of the genes composing the Cgap1 bTM.

### Pap1p as a structural model to understand the evolution of Yap1p, Cgap1p and Cap1p DNA binding properties

**Figure 4:**
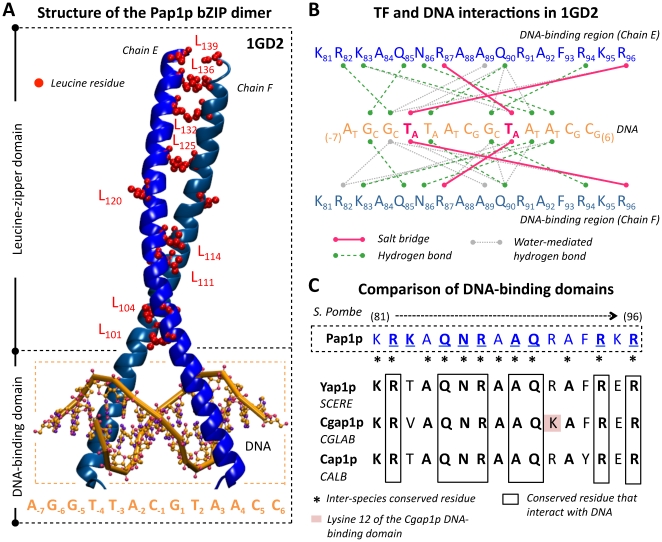
Structural explorations of yeast AP-1 transcription factor DNA recognition properties. (A) Structure of the Pap1p bZIP dimer as defined in the PDB file 1GD2. Pap1p is the closest Yap1p functional homologue in the yeast *S. pombe* (see Main Text). Two identical chains of Pap1p proteins are represented. They are labeled E and F and colored in blue. Only the leucine-zipper domains and the DNA-binding regions are shown here. They are surrounding with dashed black boxes. Leucine residues in the coiled coil region responsible for the dimerization are colored in red. The DNA fragment at which the Pap1p proteins are associated is represented in orange and is surrounding with a dashed orange box. The sequence is indicated below: AGGTTACGTAACC. Note that this sequence contains the motif TTACGTAA that is the exact YRE-A motif identified in promoter of Cgap1p-dependant genes (Figure 3). (B) Predicted interactions between Pap1p TF and DNA in the 1GD2 structure presented in (A). Three types of interactions are represented: “Salt bridge” with a pink lines, “Hydrogen bound” with a green dashed lines and “Water-mediated hydrogen bound in grey dashed lines. These interactions were identified using the MONSTER web tool (see Materials and Methods). Nine residues of the Pap1p protein interact with DNA: R_82_, K_83_, Q_85_, N_86_, R_87_, A_89_, Q_90_, R_94_ and R_96_. (C) Comparison of the DNA-binding domains of the AP-1 proteins Ypap1p (in *S. cerevisiae*), Cgap1p (in *C. glabrata*) and Cap1p (in *C. albicans*) with the DNA-binding domain of Pap1p (in *S. pombe*). Protein residues that are conserved in the four species analyzed in this study are labeled with a black star. In Pap1p protein, the 9 residues that are predicted to interact with DNA (see B) are underlined. From these 9 interacted residues, 8 are strictly conserved in other species, they are surrounding with a black box. Note that in the protein Cgap1p, the residue 12 described by Kuo *et al.* (see Main Text) is highlighted in pink.

In a final step in this analysis, we tried to connect our *de novo cis*-regulatory motif predictions with structural data related to bZIP TFs. As no structural information was available in the literature on Yap1p, Cgap1p and Cap1p proteins, binding a DNA target sequence, we considered data available on the Pap1p/DNA interaction, for which a high-resolution crystallographic structure was available (PDB code 1GD2, [13]). Pap1p is the Yap1p closest functional homologue in the yeast *S. pombe*. Like Yap1p, Cgap1p and Cap1p, Pap1p is involved in drug resistance and oxidative stress response [43]. The overall structure of the Pap1p bZIP dimer bound to the DNA sequence AGGTTACGTAACC is presented Figure 4A. The leucine-zipper domain (which mediates dimerization) and the DNA-binding domain are surrounding with dashed lines. Note that even if the yeast *S. pombe* was separated from *S. cerevisiae* and *Candida* species by a rather long evolutionary distance (at least 400 million years between *S. pombe* and *S. cerevisiae* [44]), the Pap1p structure appeared to be a relevant reference for two reasons. First, pairwise alignments between Pap1p and others yeast AP-1 TFs (Yap1p, Cgap1p, Cap1p) showed a high level of amino acid conservation, especially considering the DNA-binding domains (>80% identity, Figure 4C). Second, the DNA in the 1GD2 structure contained the sequence TTACGTAA that was the exact YRE-A motif published by Kuo *et al.* [23] and identified in the promoters of Cgap1p-dependant genes (see previous section). Therefore, the Pap1p structure represented an interesting opportunity to characterize and compare the mechanisms that underlined the binding of bZIP motifs to related but different DNA sequences. We used the MONSTER web-tool [45] to identify from this structure the potential stabilizing non-bonding interactions between residues of the DNA-binding domain of Pap1p and the DNA sequence. These interactions are represented in Figure 4B. The Pap1p/DNA crystallographic complex revealed 9 amino acids (R_82_, K_83_, Q_85_, N_86_, R_87_, A_89_, Q_90_, R_94_ and R_96_) as being engaged in salt bridges or hydrogen bonds, with either bases or phosphate groups of the specific DNA target. Interestingly, the two arginines (R_87_ and R_96_) engaged in salt bridges interacted with two thymines highly conserved in the 4 consensus sequences presented above (TTA•TAA, Figure 3). Moreover, from the 9 residues of Pap1p that interact with DNA, 8 appeared to be conserved in the Yap1p, Cgap1p and Cap1p DNA-binding domains (Figure 4C). All together, these observations suggested that the general mechanisms ensuring the specific recognition of the TTA•TAA half-sites were highly conserved in the four yeast species examined here. Finally, it should be noted that no interaction was identified between DNA and the R_91_ Pap1p residue. This arginine was conserved in Yap1p and Cap1p TFs, but was changed in a lysine in Cgap1p (see the K residue colored in pink, Figure 4C). This mutation in *trans* was proposed to be responsible for the specific DNA recognition of YRE-A motif by Cgap1p [23]. The Pap1p/DNA structure challenged this interpretation since it demonstrated that, although Pap1p, like Yap1p and Cap1p, had an arginine in position 91, it was able, like Cgap1p, to have a stable interaction with a YRE-A motif. Finally, an interesting feature of each DNA consensus identified *de novo* from the promoter sequences of the bTM-genes relied on the presence of an adenine (more rarely a cytosine) in 5′ of the canonical YREs (Figure 3). Our analyses of the 1GD2 structure showed that the arginine R_82_ forms a hydrogen bond with the base just before the TTA segment (Figure 4B). Also, a non-specific hydrogen bond was established between the glutamine Q_85_ and one more external phosphate group (Figure 4B). These interactions extended the Pap1p/DNA interface beyond the canonical TTA•TAA half sites, the basic part of Pap1p extensively filling and interacting with the DNA major groove. Since R_82_ and Q_85_ were conserved in Yap1p, Cgap1p and Cap1p, one can reasonably suppose that these proteins also covered a DNA segment larger than strictly the TTA•TAA half-sites, which gives credence to the functional significance of the supplementary adenine found in this study (Figure 3).

## Discussion

Comparative functional analyses have been made possible by the accumulation of large-scale gene expression datasets for an increasing number of organisms [46], [47]. Until recently, standard approaches for comparing genome-wide expression data between yeast species relied essentially on protein sequence alignments defining orthologous relationships between genes and functional annotation transfers from the model yeast *S. cerevisiae* [48], [49], [50], [51], [52]. These approaches gave valuable results, but the genetic tractability of more and more yeast species now allows to directly investigate the regulatory relationships between genes among species. In this work we proposed a suite of procedures to *(i)* reconstruct TMs from heterogeneous genome-wide functional datasets (microarray experiments in wild type and mutant strains, ChIP-chip analyses, Figure 1) and *(ii)* exploit these TMs in terms of *de novo cis*-regulatory motif analyses (Figure 3). Our rationale was to select, in each species, experimental information obtained in identical physiological conditions (benomyl induced-stress) and to combine, at each step of the procedure, the results obtained with several up-to-date bioinformatics methodologies, with complementary advantages and limitations (SAM, LIMMA, SMVar, ChIPmix, SCOPE, Oligo-Analysis, MEME, MONSTER).

Using this procedure, we inferred in three yeast species the “benomyl-specific TMs (bTMs)” associated to the AP-1 orthologous TFs Yap1p (in *S. cerevisiae*), Cgap1p (in *C. glabrata*) and Cap1p (in *C. albicans*). Remarkably, we observed that only a small number of genes shared orthologous relationships between the three bTMs (∼15%, Figure 2A). This apparently low conservation is consistent with published reports analyzing the evolution of various transcriptional pathways mediated by the TFs Ste12p [53], Mcm1p [54] or Yap1p [23]. Such an observation questions the widely used methodology that consists in defining the function of newly sequenced *Candida* genes using Gene Ontology annotation transfer *via* orthologous or homologous relationships with gene in *S. cerevisiae* [52]. It is clear that such an approach can be hazardous and may provide high rates of false positive and false negative genes (>70% in case of AP-1 bTMs, Figure 2B). Still, the TFs Yap1p, Cgap1p and Cap1p play very similar roles in cellular redox homeostasis [14], [16], [17]. Careful inspection of the genes identified in each bTMs provided insight in the evolutionary mechanisms involved in both the rewiring of the yeast AP-1 bTMs and the maintenance of their key functions. For instance, many genes in the Cgap1p and Cap1p bTMs share orthologous relationships with Yap1p-dependent genes, which respond to other oxidative sources than benomyl. It has been shown that the protein Yap1p can control the transcription of different sets of genes, depending on the origin of the oxidative stress and on the subsequent post-translational modifications of Yap1p [28]. We can reasonably postulate that this post-translational level of regulation was also subjected to modification during evolution. This resulted in our observation that orthologous genes conserved their transcriptional control by AP-1 TFs in each species, but respond to different stimulus. Gene duplications and multigenic protein families are other parameters that can explain apparent changes of the yeast AP-1 bTMs. This is nicely illustrated by the OYE genes, which encode NADPH oxydoreductases involved in sterol metabolism, oxidative stress response, and programmed cell death. In *S. cerevisiae*, only two OYE genes (OYE2 and its paralogue OYE3) belong to the Yap1p bTM, whereas in *C. glabrata* and *C. albicans* respectively, 4 and 3 OYE paralogues are responding to benomyl under the control of Cgap1p or Cap1p TFs (Dataset S1). In the three yeasts, the general function mediated by the OYE genes is therefore conserved, but because of several duplication events, clear orthologous relationships between genes are difficult to assign. Additionally, AP-1 proteins belong, in each species, to yeast activator protein (Yap) families, which is composed of 3 to 8 paralogous genes in *Hemiascomycetes*. In *S. cerevisiae*, this family comprises eight members (Yap1p to Yap8p) that carry both overlapping and distinct biological functions [28], and which recognizes similar DNA consensus [55]. Since these factors have been shown to interact functionally and possibly, physically [55], they certainly cross-influenced the evolution of their respective TMs. In *C. glabrata* and *C. albicans*, only 7 and 4 members were identified, respectively (see Text S5). This lower number of AP-1 TFs in *C. albicans* is connected to the whole genome duplication that arose in the common history of *S. cerevisiae* and *C. glabrata*, but not in the *C. albicans* ancestors. For this parameter also, the context of *C. glabrata* is closer to *S. cerevisiae* than *C. albicans* is. Still, the properties of Yap1p seem to be closer to Cap1p than those of Cgap1p. This underlines the fact that the evolution of regulatory networks does not necessarily follow the phylogeny of genomic sequences. Noteworthy, more than half of the genes that belong to the Cgap1p and Cap1p bTMs defined in this study, exhibit orthologous genes in *S. cerevisiae* for which no functional relationship with the TF Yap1p is described. Further experimental analyses will be needed to validate the potential role of these genes in the response to benomyl induced-stress, but the YRE consensus motifs observed in their promoter sequences argues in favor of their actual regulation by TFs Cgap1p and Cap1p.

**Figure 5:**
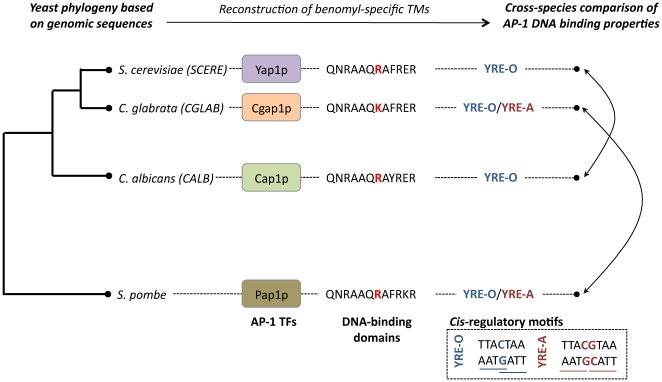
Evolution of the DNA binding properties of the yeast AP-1 transcription factors. Tree symbolizing the evolutionary distances between the four yeast species considered in this study is presented. Note that the lengths of the branches do not represent rigorous quantifications of the evolutionary distances. The names of the Yap1p orthologous proteins in each species are represented in colored boxes. The protein sequences of the basic region of their DNA binding domains are indicated for each factor together with the DNA consensus type (YRE-O or YRE-A). The amino acid in position 12, which had been hypothesized to be a key determinant of the discrimination between YRE-A and YRE-O recognizing factors (see the Main Text), has been highlighted in red. More precisely, the *cis*-regulatory motif consensus for Yap1p and Cap1p TFs is MTKASTMA (this study), the *cis*-regulatory consensuses identified for Cgap1p are MTTASSTAA and ATTACHAAW (this study) and the DNA consensuses identified for Pap1p are TTACGTAA and TTACTAA [13].

Obviously, the evolution of transcriptional regulatory networks is tightly connected to the evolution of the TF/DNA binding properties. Our *de novo cis*-regulatory motif analyses allowed us to observe an impressive conservation in the sequences identified from the promoters of Yap1p- and Cap1p-dependent genes (Figure 3). These sequences are YRE-O motifs [23] and include the classical BRE [19]. Obtaining such identical results analyzing the promoter sequences of genes whose coding sequences, as stated above, are not particularly conserved (Figure 2) gives credence to our *in silico* predictions of bTMs (Figure 1). In a recent study, Kuo *et al.* [23] proposed that whereas Yap1p recognized exclusively YRE-O motifs, Cgap1p prefers YRE-A. To explain this difference, they proposed an interesting model involving compensatory *cis* and *trans* mutations between DNA sequence and the Cgap1p protein. Indeed, unlike Yap1p and Cap1p proteins, the DNA binding domain of Cgap1p protein exhibits the replacement of an arginine by a lysine in position 12 of the basic region (residue K, Figure 4C). Several Yap1p paralogous proteins like Yap3p, Yap4p or Yap6p also prefer YRE-A rather the YRE-O motifs and, like Cgap1p, exhibit a lysine in position 12 [23], [55]. Kuo *et al.* [23] therefore suggested a strict dichotomy between the yeast AP-1 proteins that recognize YRE-O motifs and the ones that bind YRE-A sequences. Our *de novo cis*-regulatory motif analyses based on the combination of *(i)* transcriptome analyses in wild type and mutant strains, *(ii)* ChIP-chip results and *(iii)* available structural data of TF/DNA interactions, only partially agree with this evolutionary model. Indeed, our systematic searches of the consensus MTTASSTAA (Figure 3) in promoters of Yap1p- and Cap1-dependant genes confirmed that YRE-A motifs were not over-represented (p-values>0.01) in the target promoters of these two TFs. However, our analyses strongly suggest that Cgap1p is actually able to recognize both the YRE-A and some variants of the YRE-O motifs and that the evolutionary divergence in the *cis*-regulatory motifs associated to this TF is less clear-cut than suggested in [23]. Notably the YRE-O consensus ATTACHAAW identified in promoters of Cgap1p-dependent genes appeared to be functionally relevant since *(i)* it included the sequence TTACAAA that was shown experimentally to act as a BRE in *C. glabrata* [21] and *(ii)* it was located in 31% of Cgap1p-dependant gene promoters, *i.e.* a percentage higher than this of the YRE-A motif MTTASSTAA (24%). Although original at the scale of the bZIP family of proteins, this particular DNA binding property of Cgap1p is not unique. It was demonstrated that the TF Pap1p in yeast *S. pombe* is able to bind *in vitro* to both YRE-O and YRE-A sequences and that both sites are active *in vivo* [13], [43]. Remarkably, Pap1p, like Yap1p and Cap1p, has a DNA binding domain that contains an arginine in position 12 (residue R_91_) of the basic region (Figure 4C and Figure 5). This information, together with our observation that this arginine does not interact directly with DNA in the crystallographic data available for Pap1p (Figure 4B), strongly suggest that the replacement of an arginine with a lysine at this position is not the only reason for the divergence of the Cgap1p DNA binding properties. Others mutations in the DNA binding domain of AP-1 proteins (for instance residue V83 in *C. glabrata*), but also possibly in other parts of the proteins, may have modified the tolerance of the Cgap1p and Pap1p TFs, hence allowing their interaction with both 7 (YRE-O) and 8 (YRE-A) base pair YREs. This model is supported by the observation that no specific interaction was identified between the protein Pap1p and the middle cytosine (TTACGTAA) of its DNA target sequence (Figure 4A and B).

An intriguing finding of our study is the presence of a supplementary adenine in 5′ position of all the BRE consensuses predicted using promoter sequences of genes in Yap1p, Cgap1p and Cap1p bTMs (Figure 3). This was previously reported for Cap1p based on *in silico* analyses [16] and for Yap1p based on an *in vitro* screening using protein binding microarrays [56]. Observing such an extension of *cis*-regulatory motifs in four different BRE consensuses, obtained independently in three different yeast species separated by 300 million years of evolution, largely support its functional significance. Available structural data also showed that the Pap1p/DNA interactions include bases that are flanking the *sensu stricto* YRE motif. Moreover, the presence of an adenine in 5′ of the consensus sequence has been described for several other sub-families of bZIP TFs, including the mammalian C/EBP (which recognize the YRE-A like motif ATTGCGCAAT) and AP1 (which recognize the YRE-O like motif ATGACTCAT) TFs.

In conclusion, this analysis revealed the complexity of the evolution of the DNA binding properties of yeast AP-1 proteins. The high conservation of the DNA binding properties of Yap1p and Cap1p proteins on one hand, and the divergence of the DNA binding properties of Cgap1p that remind properties of Pap1p on the other hand, is non-intuitive considering that *C. glabrata* is much more closely related to *S. cerevisiae* than *C. albicans* [57] and that *S. pombe* is between 300 and 1000 million years distant from the three other species [44]. The case study of AP-1 proteins nicely demonstrates that the evolution of transcriptional networks does not necessarily follow the global conservation of genomic sequences and the species phylogeny (Figure 5). A challenging question would be to understand the actual properties of the common ancestor of all the yeast AP-1 proteins. The experimental determination of the DNA motifs recognized by the paralogous proteins of Cgap1p and Cap1p could certainly help in the reconstruction of the evolutionary path followed by each of these proteins. Also our analysis questions our ability to understand the molecular basis of the genomic response to stress in *C. glabrata*, which is an emerging opportunistic human pathogen, by transferring functional evidences obtained in *S. cerevisiae* and *C. albicans*. Still, the physiological role of the TFs Yap1p (in *S. cerevisiae*), Cgap1p (in *C. glabrata*), Cap1p (in *C. albicans*) and Pap1p (in *S. pombe*), in response to oxidative stress is conserved, despite important rewiring in their lists of target genes. This provides another proof that, in yeasts, selective pressures on phenotypic traits can deal with extensive rearrangements in the underlying regulatory networks.

## Materials and Methods

### Experimental datasets

Microarray analyses of the transcriptome responses of *S. cerevisiae*, *C. glabrata* and *C. albicans* following similar treatments with the antifungal agent benomyl were obtained from the work of Lelandais *et al.* [58] and Znaidi *et al.* [16]. The raw data were collected from the Gene Expression Omnibus (GEO) database [59], under the accession number GSE10244 and GSE14258. The resulting expression matrices comprised information for 4986 genes in *S. cerevisiae*, 5771 in *C. glabrata* and 4583 in *C. albicans*. Microarray datasets for the benomyl response of strains deleted for the AP-1 TFs were collected from the studies of Lucau-Danila *et al.* [6] (*ΔYAP1*), Lelandais *et al.* [21] (*ΔCgAP1*) and Znaidi *et al.* [16] (*ΔCAP1*). The resulting expression matrices comprised data for 6189 genes in *S. cerevisiae*, 5196 in *C. glabrata* and 4974 in *C. albicans*. ChIP-chip datasets for each of the three AP-1 TFs were obtained from the works of Salin *et al.* [18] (Yap1p, upon request to the authors), Kuo *et al.* [23] (Cgap1p, GEO database under accession number GSE15818) and Znaidi *et al.* [16] (Cap1p, GEO database, accession number: GSE15104). The resulting matrices comprised data for 13.824 probes in *S. cerevisiae*, 41.799 in *C. glabrata* and 66.555 in *C. albicans*.

### Identification of differentially expressed genes

To identify the genes whose expression was significantly modified in response to benomyl addition or in response to the deletion of one of the yeast AP-1 TF, three different algorithms were applied: Significance Analysis of Microarrays (SAM) [29], Linear Models for MicroArray data (LIMMA) [30] and Structural Model for Variances (SMVar) [31]. These algorithms were chosen because they were representative of different variance modeling strategies in gene expression data [60]. Default parameters were used during algorithm runs and differentially expressed genes were selected based on a FDR value lower than 5% (for SAM) or p-values lower than 5% (for LIMMA and SMVar). Finally, we considered only those genes that were identified as differentially expressed by at least two different algorithms.

### Identification of yeast AP-1 transcription factor binding sites *in vivo*

To identify the promoter of genes cross-linked with one of the AP-1 TFs (Yap1p, Cgap1p or Cap1p), we used the ChIPmix methodology [61]. Compared to SAM, LIMMA and SMVar methods that work on log ratio, ChIPmix has the originality to directly analyze the signals of IP (DNA fragments cross-linked to TF protein) and INPUT (genomic DNA) by modeling the distribution of the IP signal conditional to the INPUT signal [32]. Default parameters were used during algorithm runs with a risk α lower than 5%. ChIPmix results were combined with those obtained using the differential analysis approach (see previous section). Only promoters of genes that were identified as differentially enriched between two immunoprecipitated DNA samples *i.e.* interest DNA (IP) and genomic DNA (INPUT) were selected. Finally, we considered as target gene for one of the AP-1 TF, those genes that were selected by two different methodologies (differential analysis and ChIPmix).

### Source of sequence data

Complete genome sequences for *S. cerevisiae* and *C. glabrata* were respectively downloaded from the Saccharomyces Genome Database (SGD) [62] and Génolevures [57] websites. For *C. albicans*, the original assembly 21 of the genome was used as described in the Candida Genome Database (CGD) [63] website. Promoter sequences located upstream from the Open Reading Frame (ORF) were obtained from the Regulatory Sequence Analysis Tools (RSAT) website [64].

### Orthology and homology assignements

The INPARANOID software [36] was used with the default parameters, to search for one-to-one orthologous relationships between genes of the three yeast genomes. 4474 orthologous genes were identified between *S. cerevisiae* and *C. glabrata*, 3733 between *S. cerevisiae* and *C. albicans* and 3621 between *C. glabrata* and *C. albicans*. Homology relationships between proteins were inferred aligning all pairs of protein sequences between two yeast genomes using the BLAST algorithm [65]. Two proteins were considered as “homologues” if *(i)* their BLAST E-value was less than 10^−2^; *(ii)* their alignment length was greater than 100 amino acids and *(iii)* the percentage identity between two sequences was greater than 25%.

### Search for *cis*-regulatory motifs in promoter sequences of genes

*De novo* motif searches were performed using three different programs: *(i)* the Suite for Computational Identification of Promoter Elements (SCOPE) program [40], *(ii)* the oligo-analysis program [41] (with a search pattern defined as 9 bases) and *(iii)* the Multiple Em for Motif Elicitation (MEME) algorithm [42]. Regulatory motifs within the promoter region of the genes were searched in upstream sequences from positions −800 to −1 (overlap with neighboring ORFs was prevented). Promoter sequences were analyzed applying these three algorithms and finally, only the regulatory patterns identified by two of the three programs were retained. We assessed whether identified motifs were observed at a frequency greater than expected by chance, by calculating p-values as described in [66] (hypergeometric distribution). A motif was considered as significantly enriched if the calculated p-value is lower than 10^−5^. A detailed illustration of the global procedure for regulatory motifs identification is presented in Text S3.

### Identification of interactions between Pap1p TF and DNA bases

Identification of the interactions between Pap1p TF and the DNA bases was performed analyzing the 1GD2 structure with the MONSTER web-tool [45]. For identification of interactions, we used a distance cut-off between 2–5 Angstroms (Å). Only interactions between residues of the DNA-binding domain and the DNA sequence were considered.

## Supporting Information

Dataset S1 **Table listing the genes identified in the AP-1 bTMs, in** ***S. cerevisiae*** **,** ***C. glabrata*** **and** ***C. albicans*** **yeast species.**
(XLS)

Dataset S2 **Table listing homologous proteins between the yeast species** ***S. cerevisiae*** **,** ***C. glabrata*** **and** ***C. albicans*** **.**
(XLS)

Text S1 **Table presenting a comparison of the list of genes identified in this study (** **Figure 1** **), with these presented in the original studies.**
(PDF)

Text S2 **Text document with detailed justifications of the criterion choice to select genes in the final AP-1 bTMs.**
(PDF)

Text S3 **Figure describing the procedure used in this study to identify** ***de novo cis*** **-regulatory motifs in promoter sequences of genes that belong to the AP-1 bTMs.**
(PDF)

Text S4 **Text document with tables presenting the detailed results of the** ***de novo cis*** **-regulatory motif search.**
(PDF)

Text S5 **Table presenting the different TFs that belong to the Yap family in yeast** ***S. cerevisiae*** **,** ***C. glabrata*** **and** ***C. albicans*** **.**
(PDF)
