# Supplementary material for: The Reconstruction of Condition-Specific Transcriptional Modules Provides New Insights in the Evolution of Yeast AP-1 Proteins
Source: PLoS One. 2011 Jun 9;6(6):e20924. doi: 10.1371/journal.pone.0020924 (PMC3111461; doi:10.1371/journal.pone.0020924)
Supplement: Text S1 — Table presenting a comparison of the list of genes identified in this study ( Figure 1 ), with these presented in the original studies. (PDF) [file pone.0020924.s003.pdf]

Text S1. Comparative analysis of the results obtained in this study with the results presented in the original publications.

We performed a comparative analysis of the results obtained in this study with the results presented in the original publications (when they were available). The results are presented Table S1 (below). The differences between our work and the original publications are entirely due to the differences in the methodologies used for raw data analyses. This nicely points out the importance of using exactly the same approach when performing cross-species comparisons.

	**SCERE**		**CGLAB**		**CALB**	
	**Original publication**	**This study**	**Original publication**	**This study**	**Original publication**	**This study**
**STEP 1**: Genome-wide expression data	228 genes	786 genes	272 genes	327 genes	432 genes	337 genes
	**-- 223 --**		**-- 234 --**		**-- 175 --**	
**STEP 2**: Mutant analyses	Not available	33 genes	118 genes	134 genes	232 genes	168 genes
			**-- 102 --**		**-- 11 --**	
**STEP 3**: ChIP chip experiments	Not available	260 genes	Not available	416 genes	140 genes	373 genes
					**-- 114 --**	

Table S1: Comparison of the genes selected in our analysis at each step of the procedure, with the genes identified in the original publications.

In *S. cerevisiae* (SCERE) and *C. glabrata* (CGLAB), our re-analyses of the raw microarray datasets allowed us to identify more genes than the original publication [2]. Notably, almost all the genes previously described were included in our list. Such a result can be explained by the use of only one algorithm in the original publication (SAM methodology), whereas we used three different methodologies (SAM, LIMMA and SMVar, see Methods in the main Text). Typically, additional genes identified here were selected with the LIMMA and/or SMVar algorithms.

The re-analysis of the ChIP-chip data in *C. albicans* (CALB) leads to a similar observation, *i.e.* more genes were identified in our study. Again, this result can be explained by the use of a unique methodology in the original publication [1] (Tilescope program [3]), whereas we used four different algorithms (SAM, LIMMA, SMVar and ChIPmix). On the other hand, our re-analysis of the transcripome datasets (wild-type and mutants strains) identified less genes than the

original publication [1]. Also, the intersections between gene lists comprised relatively few genes. This observation can be explained by the methodology used by the authors in their original work. They applied a simple log fold change criterion to select differentially expressed genes. This approach gave interesting results [1], but has the important limitation not to take into account the experimental variability related to technical replicates, and is therefore more likely to select genes with artefacts in expression variations. SAM, LIMMA and SMVar approaches were developed precisely to optimize the variability estimation and preferentially selected genes based on the reproducibility of their expression measurements. Still, the two approaches converged on a set of core targets of Cap1p-dependant genes (e.g. MDR1, GCS1, YCF1, ...).

References

1. Znaidi, S., et al., *Identification of the Candida albicans Cap1p regulon.* Eukaryot Cell, 2009. **8**(6): p. 806-20.
2. Lelandais, G., et al., *Genome adaptation to chemical stress: clues from comparative transcriptomics in Saccharomyces cerevisiae and Candida glabrata.* Genome Biol, 2008. **9**(11): p. R164.
3. Zhang, Z.D., et al., *Tilescope: online analysis pipeline for high-density tiling microarray data.* Genome Biol, 2007. **8**(5): p. R81.
