# Supplementary material for: The Reconstruction of Condition-Specific Transcriptional Modules Provides New Insights in the Evolution of Yeast AP-1 Proteins
Source: PLoS One. 2011 Jun 9;6(6):e20924. doi: 10.1371/journal.pone.0020924 (PMC3111461; doi:10.1371/journal.pone.0020924)
Supplement: Text S2 — Text document with detailed justifications of the criterion choice to select genes in the final AP-1 bTMs. (PDF) [file pone.0020924.s004.pdf]

Text S2. Justifications of the criterion choice to select genes in the final AP-1 bTMs.

To identify the transcriptional modules (TMs) related to the transcription factors (TFs) Yap1p (in *S. cerevisiae*), Cgap1p (in *C. glabrata*) and Cap1p (in *C. albicans*), we used an integrative framework that combined *(i)* multiple sources of experimental data and *(ii)* multiple bioinformatics approaches to analyze these data. To summarize, we considered in each species three different layers of information based on the analysis of genome-wide datasets. First we used expression patterns of genes to identify genes that are significantly up-regulated in response to benomyl induced-stress (see Step 1, Figure 1). Second we analyzed the transcriptome alterations in yeast strains deleted for the genes coding the yeast AP-1 TFs (respectively Yap1p, Cgap1p and Cap1p) (see Step 2, Figure 1), and third we searched for genomic locations of the TF binding sequences using ChIP-chip experiments (see Step 3, Figure 1). Note that each experimental dataset was carefully chosen in order to ensure both intra and inter-species comparisons of the obtained results, *i.e.* comparable benomyl doses in each species and similar time point measurements for transcriptome analyses.

Results obtained in Step 1, 2 and 3 were finally combined (see Step 4, Figure 1). Genes selected in “Step 1 and Step 2”, or in “Step 1 and Step 3” were conserved in the final AP-1 TMs. Note that the formula “Step 1 and Step 2 and Step 3” is more stringent and should reduce the risk to select false positive genes in the final transcriptional modules (TMs). However, this formula allowed the selection of only a very small number of genes in each transcriptional module (16 genes in *S. cerevisiae*, 28 genes in *C. glabrata* and 37 genes in *C. albicans*). In this situation, the advantage of reducing the risk to select false positive genes is counterbalanced by an important increase of the risk to not select interesting genes (false negative genes). In particular, a gene for which no data was available in Step 2 (mutant analyses) or Step 3 (ChIP-chip experiments) could not be selected with the criterion “Step 1 and Step 2 and Step 3”. For instance, the ChIP-chip data available for *S. cerevisiae* were obtained using microarrays, which contained only one or two probes for each intergenic region (whereas results for *C. glabrata* and *C. albicans* species arisen from tiling arrays). After data pre-processing, we noticed that no information was available for many genes (FLR1, FRM2, YCP4, ECM4, ERO1 for instance), well described in the literature as being target genes of Yap1p. Excluding these genes from our analysis only for technical reasons would have finally reduced the biological relevance of the study, and the choice of the formula “(Step 1 and Step 2) or (Step 1 and Step 3)” was fully supported by the significance of the results that we obtained in the subsequent analyses.
