# Supplementary figures and images for: The Reconstruction of Condition-Specific Transcriptional Modules Provides New Insights in the Evolution of Yeast AP-1 Proteins
Source: PLoS One. 2011 Jun 9;6(6):e20924. doi: 10.1371/journal.pone.0020924 (PMC3111461; doi:10.1371/journal.pone.0020924)

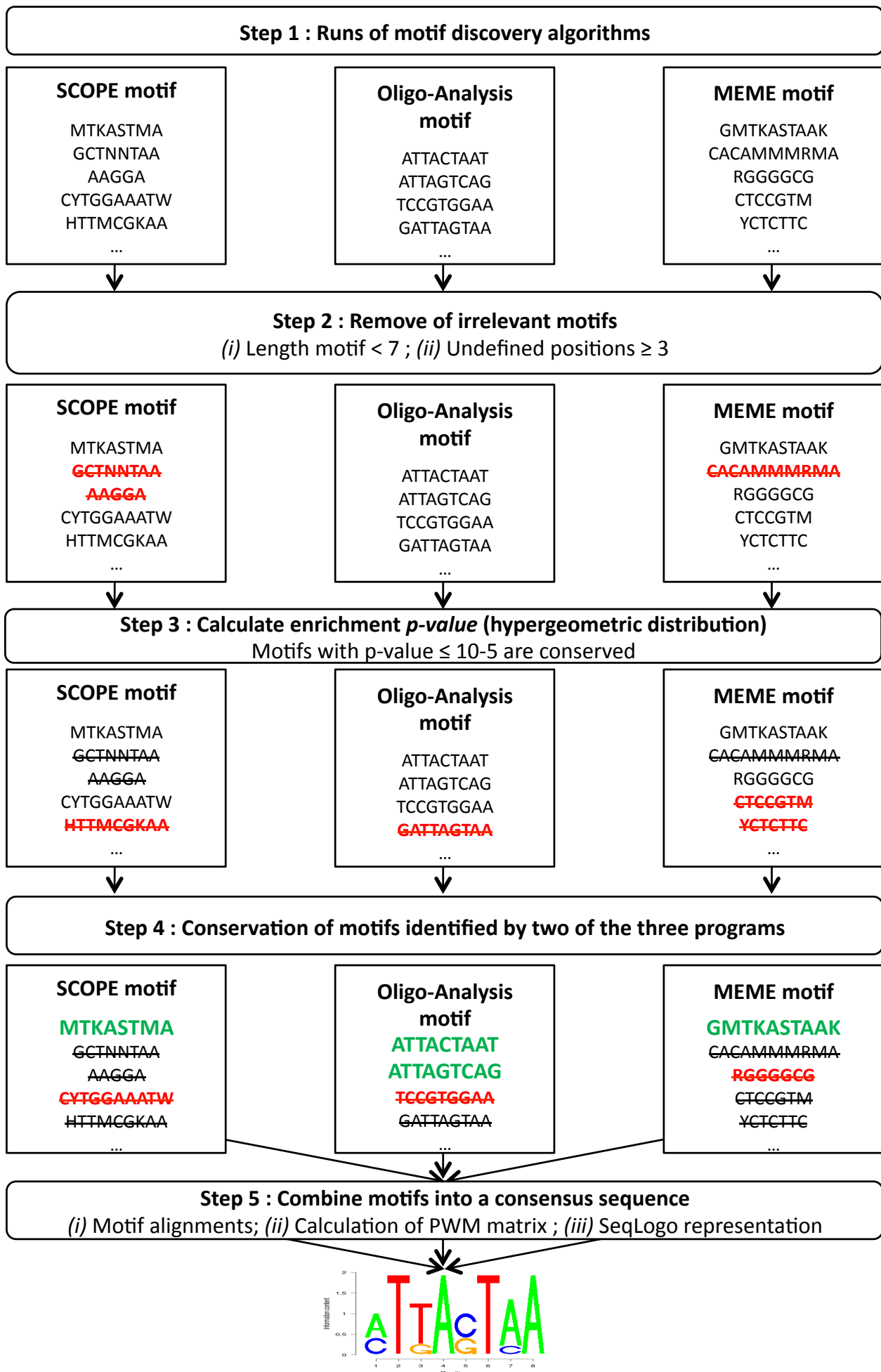

Supplement: Text S3 — Figure describing the procedure used in this study to identify de novo cis -regulatory motifs in promoter sequences of genes that belong to the AP-1 bTMs. (PDF) [file pone.0020924.s005.pdf]
