# Supplementary material for: The Reconstruction of Condition-Specific Transcriptional Modules Provides New Insights in the Evolution of Yeast AP-1 Proteins
Source: PLoS One. 2011 Jun 9;6(6):e20924. doi: 10.1371/journal.pone.0020924 (PMC3111461; doi:10.1371/journal.pone.0020924)
Supplement: Text S4 — Text document with tables presenting the detailed results of the de novo cis -regulatory motif search. (PDF) [file pone.0020924.s006.pdf]

Text S4. Complete list of *cis-r*egulatory motifs identified in the study

To detect significant *de novo cis*-regulatory motifs in promoter sequences of genes whose expression was regulated by yeast AP-1 TFs during benomyl stress response, we used the global procedure described in the main text and illustrated in the Text S3. The finally conserved *de novo cis*-regulatory motifs are shown below:

Motif[a]	Algorithm[b]	%[c]	p-value[d]
**MTKASTMA**	SCOPE	82.10	4.48E-19
**GMTKASTAAK**	MEME	38.80	1.54E-23
**ATTACTAAG**	Oligo-Analysis	14.90	9.14E-09
**CATTACTAA**	Oligo-Analysis	13.40	2.62E-07
**TATTACTAA**	Oligo-Analysis	13.40	1.14E-05
**ATTACTAAT**	Oligo-Analysis	11.90	2.05E-05
**GCTTACTAA**	Oligo-Analysis	11.90	1.26E-08
**ATTAGTCAG**	Oligo-Analysis	10.40	9.10E-08
**GCTGACTAA**	Oligo-Analysis	10.40	2.86E-07
**ATTAGTAAG**	Oligo-Analysis	10.40	6.91E-06
**ACTTAGTAA**	Oligo-Analysis	10.40	6.91E-06
**CTTACTAAG**	Oligo-Analysis	09.00	5.42E-05

Detailed *de novo cis*-regulatory motif information in *S. cerevisiae*.

[a] The motif identified and preserved during the search pattern. M designates A or C, K designates G or T and S designates C or G.

[b] The motif discovery algorithm that identified the motif given in the first column.

[c] The percentages of genes of the Yap1p bTM whose motif described in first column is found in their promoter.

[d] *P-values* of the representation in Yap1p bTM of motif described in first column . These have been calculated using a hypergeometric distribution.

Motif[a]	Algorithm[b]	%[c]	p-value[d]
**MTTASSTAA**	SCOPE	24.50	7.25E-14
**CMTTACCTAA**	MEME	9.2	4.71E-10
**ATTAGCTAA**	Oligo-Analysis	8.2	7.62E-07
**ATTAGGTAA**	Oligo-Analysis	8.2	4.07E-06
**ATTACCTAA**	Oligo-Analysis	8.2	4.07E-06
**ATTACYAAW**	MEME	19.4	6.54E-05
**ATTACAAAT**	MEME<br>Oligo-Analysis	12.2	4.65E-05

Detailed *de novo cis*-regulatory motif information in *C. glabrata*.

[a] The motif identified and preserved during the search pattern. M designates A or C, S designates C or G, Y designates T or C and W designates A or T.

[b] The motif discovery algorithm that identified the motif given in the first column.

[c] The percentages of genes of the Cgap1p TM whose motif described in first column is found in their promoter.

[d] *P-values* of the representation in Cgap1p TM of motif described in first column . These have been calculated using a hypergeometric distribution.

Note that the observation that only 50% of Cgap1p-dependant genes exhibit MTTASSTAA or ATTACHAAW motifs in their promoter sequences (see the main text) suggests that other Cgap1p potential motifs have to be discovered. In that respect, we could observe that when the consensus sequence ATTACHAAW is degenerated in sequence MTKASWMA (with M = (A or C); K = (G or T); S = (G or C); W = (A or T)), the number of Cgap1p-dependant genes that comprise this consensus in their promoter sequences increased from 31% (ATTACHAAW, see Figure 2) to 80%. Motifs that remain to be identified in Cgap1p-dependant genes are certainly YREO-like sequences included in the MTKASWMA consensus. However, this information needs to be considered with caution. Indeed, the consensus MTKASWMA is highly degenerated and therefore the enrichment p-value is 2.10-4, i.e. a value that is higher than the threshold value (10-5) that we used to select motifs in others species (see Figure 2 and Text S3 for details). All together, these data support the hypothesis that Cgap1p binding properties are more flexible and permissive than the one of Yap1p and Cap1p.

**Motif**[a]	**Algorithm**[b]	**%**[c]	**p-value**[d]
**HNMTKASTMAK**	SCOPE	57.70	1.74E-34
**ATTACTAAK**	MEME	38.50	2.83E-31
**ATTWGTMAKC**	MEME	37.70	6.50E-40
**ATTACTAAT**	Oligo-Analysis	32.30	1.79E-27
**GATTACTAA**	Oligo-Analysis	17.10	3.10E-17
**AATTAGTAA**	Oligo-Analysis	14.60	1.18E-07
**GATTAGTAA**	Oligo-Analysis	12.30	3.66E-10
**GCTGACAAA**	Oligo-Analysis	09.20	3.61E-11

Detailed *de novo cis*-regulatory motif information in *C. albicans*.

[a] The motif identified and preserved during the search pattern. H designates A or C or T, N designates any bases, M designates A or C, K designates G or T, S designates C or G and W designates A or T.

[b] The motif discovery algorithm that identified the motif given in the first column.

[c] The percentages of genes of the Cap1p TM whose motif described in first column is found in their promoter.

[d] *P-values* of the representation in Cap1p TM of motif described in first column . These have been calculated using a hypergeometric distribution.
