# Supplementary material for: The Reconstruction of Condition-Specific Transcriptional Modules Provides New Insights in the Evolution of Yeast AP-1 Proteins
Source: PLoS One. 2011 Jun 9;6(6):e20924. doi: 10.1371/journal.pone.0020924 (PMC3111461; doi:10.1371/journal.pone.0020924)
Supplement: Text S5 — Table presenting the different TFs that belong to the Yap family in yeast S. cerevisiae , C. glabrata and C. albicans . (PDF) [file pone.0020924.s007.pdf]

Text S5. Description of the Yap family in yeast species *S. cerevisiae*, *C. glabrata* and *C. albicans.*

To identify the members of the Yap family in each yeast species, we used a general strategy comparable to this presented in [1]. Results obtained in *C. glabrata* and *C. albicans* were also manually inspected. In *C. glabrata* seven members were identified, whereas in *C. albicans* only 4 members were found (see below). This lower number of AP-1 transcription factors in *C. albicans* is connected to the whole genome duplication that arose in the common history of *S. cerevisiae* and *C. glabrata*, but not in the *C. albicans* ancestors.

Gene name	Description
Saccharomyces cerevisiae	
YML007W|YAP1	Basic leucine zipper (bZIP) transcription factor required for oxidative stress tolerance; activated by H2O2 through the multistep formation of disulfide bonds and transit from the cytoplasm to the nucleus; mediates resistance to cadmium
YDR423C|YAP2	AP-1-like basic leucine zipper (bZIP) transcriptional activator involved in stress responses, iron metabolism, and pleiotropic drug resistance; controls a set of genes involved in stabilizing proteins; binds consensus sequence TTACTAA
YHL009C|YAP3	Basic leucine zipper (bZIP) transcription factor
YOR028C|YAP4	Basic leucine zipper (bZIP) transcription factor of the yAP-1 family, mediates pleiotropic drug resistance and salt tolerance; nuclearly localized under oxidative stress and sequestered in the cytoplasm by Lot6p under reducing conditions
YIR018W|YAP5	Basic leucine zipper (bZIP) transcription factor
YDR259C|YAP6	Putative basic leucine zipper (bZIP) transcription factor; overexpression increases sodium and lithium tolerance; computational analysis suggests a role in regulation of expression of genes involved in carbohydrate metabolism
YOL028C|YAP7	Putative basic leucine zipper (bZIP) transcription factor
YPR199C|YAP8	Transcriptional activator of the basic leucine zipper (bZIP) family, required for transcription of genes involved in resistance to arsenic compounds
Candida glabrata	
CAGL0H04631g	similar to uniprot|P19880 Saccharomyces cerevisiae YML007w YAP1 (ohnolog of YDR423C) Basic leucine zipper (bZIP) transcription factor required for oxidative stress tolerance

CAGL0F03069g	some similarities with uniprot|P24813 Saccharomyces cerevisiae YDR423c CAD1 (ohnolog of YML007W) AP-1-like basic leucine zipper (bZIP) transcriptional activator involved in stress responses, iron metabolism, and pleiotropic drug resistance
CAGL0K02585g	some similarities with uniprot|P38749 Saccharomyces cerevisiae YHL009c YAP3 Basic leucine zipper (bZIP) transcription factor
CAGL0M10087g	some similarities with uniprot|P38749 Saccharomyces cerevisiae YHL009c YAP3 Basic leucine zipper (bZIP) transcription factor
CAGL0M08800g	weakly similar to uniprot|Q03935 Saccharomyces cerevisiae YDR259c YAP6 (ohnolog of YOR028C) Putative basic leucine zipper (bZIP) transcription factor
CAGL0F01265g	some similarities with uniprot|Q08182 Saccharomyces cerevisiae YOL028c YAP7 (ohnolog of YIR018W) Putative basic leucine zipper (bZIP) transcription factor
CAGL0K08756g	weakly similar to uniprot|P40574 Saccharomyces cerevisiae YIR018w YAP5 (ohnolog of YOL028C) Basic leucine zipper (bZIP) transcription factor
Candida albicans	
orf19.1623|CAP1	Transcription factor, AP-1 family; role in oxidative stress response and resistance, multidrug resistance; oxidative stress regulates nuclear localization; partially complements S. cerevisiae yap1 mutation; human neutrophil-induced
orf19.3193|FCR3	Transcriptional regulator of the bZip family; partially functionally complements the fluconazole sensitivity of an S. cerevisiae pdr1 pdr3 double mutant; probable ortholog of S. cerevisiae Yap3p
orf19.681|HAP43	CCAAT-binding factor (CBF)-dependent transcription factor required for iron-limitation response; similar to transcription factor AP-1; transcription is negatively regulated by Sfu1p; ciclopirox olamine induced
orf19.861	Predicted transcriptional regulator with bZip domain; possibly an essential gene, disruptants not obtained by UAU1 metho

Gene descriptions were obtained from the following websites : SGD http://www.yeastgenome.org/ (for S. cerevisiae), Genolevures http://www.genolevures.org/ (for C. glabrata) and CGD http://www.candidagenome.org/ (for C. albicans).

Reference

1. Fernandes, L., C. Rodrigues-Pousada, and K. Struhl, *Yap, a novel family of eight bZIP proteins in Saccharomyces cerevisiae with distinct biological functions.* Mol Cell Biol, 1997. **17**(12): p. 6982-93.
